# Gum Lancing in Difficult Primary Dentition

**Published:** 1894-07

**Authors:** E. C. Kirk


ARTICLE VII.

### GUM LANCING IN DIFFICULT PRIMARY DENTITION.

BY DR. E. C. KIRK, D. D. S.

An address before the Woman's Dental Association of the United States.

I should feel myself constrained to offer you an apology for bringing to your notice such an antiquated topic, were it not that old as it is the vexed question of its legitimacy is still in dispute, and the procedure has not gained general acceptance among medical and dental practitioners. It is not my purpose to enter into any historical resume of the views entertained by various writers of greater or less prominence, who have recorded their opinions from the times of Hippocrates and Galen to the present time; but in this paper to call your attention to the main differences in the two principal schools of thought on this subject, and urge upon you the necessity of investigating it for yourselves, not from the point of view of the medical practitioner, but from the background of the special training and culture which you possess as dentists. I think we may safely dismiss from consideration the views and theories of earlier medical writers upon this subject, and confine ourselves wholly to an investigation of the more recent, for in a certain and more or less definite degree the former are included in the latter—at least so much of the former as has been considered valuable. The question of gum lancing in difficult primary dentition has been the subject of much animated discussion during the past eighteen months. Especially since the publication of a book by Dr. Forchheimer of Cincinnati, Ohio, on "Disease of the mouth in children" (non-surgical), in May, 1892, in which work the author took most positive ground against the operation as a therapeutic measure for the relief of diseases incident to the teething period. His conclusions respecting the operation were tersely stated as follows:

1. It is useless—(a) as far as giving relief to symptoms; (b) as far as facilitating or hastening teething.

2, It is useful only as blood-letting, and ought to be used as such.

3. It is harmful—(a) in producing local trouble; (b) in producing general disturbances on account of hemorrhage; (c) in having established a method which is too general to do specific good, and too specific for genera use.

4. It is to be used only as a surgical procedure to give relief to surgical accidents.

I have quoted these conclusions at length, because they fairly represent the opinions, and are arguments generally put forward by that class of medical practitioners who do not know anything about the operation from practical experience, and still less from an intelligent understanding of the rationale of the procedure.

A critical review of Forcheimer's book appeared in the

*Dental Cosmos* for June, '92, pointing out some of the fallacies that were apparent in his conclusions, and advocating the operation of gum lancing on rational grounds from the standpoint of accepted knowledge of the anatomy, physiology, and nervous relationships of the tissues and organs involved. The subject was further discussed by numerous authors and editors in medical and dental periodicals, and finally Dr. Magitot presented it in a communication to the French Academy of Medicine. In this paper the author took the ground that inasmuch as dentition was a purely physiological process, there could be no such things as "accidents of dentition," or, as we express it in this country, diseases incident to or dependant upon dentition. His argument to sustain his position was, like that of Forchheimer, based solely upon analytical reasoning from premises which could not be accepted by any one conversant with the clinical aspects of the subject.

As a sequel to this discussion of the French Academy, M. Poinsot, one of the participants, has elaborated the subject in an interesting volume recently published, entitled, "Accidents of the first dentition."

It will be seen, then, that the class of practitioners who are antagonistic to the operation of gum lancing are those who, like Forchheimer, object to it because they do not understand why it shohld be done, nor how to do it—mistaking gum scarification for gum lancing,—and those who, like Magitot, oppose the operation as a therapeutic measure because dentition is a physiological process: *ergò*, there can be no diseases due to or caused by it; hence lancing the gums for the relief of any disorders intercurrent with dentition is irrational and unnecessary. During the past twenty years it has been my lot to have been somewhat closely related to medical as well as dental work, and to have had rather frequent opportunities to observe cases of difficult dentition, and the effect of an intelligent use of the gum lancet as a therapeutic measure for the relief of disorders incident thereto; hence my faith in the efficacy of the operation is the outgrowth of personal experience as well as observation, and if I shall seem to advocate it somewhat dogmatically, it is because I am convinced that the facts sustain my belief.

The argument of Magitot and his followers, it seems to me, is easily demolished after an investigation of his major premise, viz.: that dentition is a purely physiological process. The answer to this is simply that dentition, while it is generally a physiological process, is not always so, and like all physiological processes, if interrupted or interfered with, may become pathological in its expression. We have only to call to mind the many accidents and fatal pathological phenomena which may attend parturition to find sufficient proof of the utter fallacy of Magitot's proposition.

The proruption of a tooth is a complex process, and includes a number of factors which, when they proceed harmoniously, produce no untoward results, and the teething process in its physiological expression is unattended with disturbances to the health status of the infant. A perfectly normal process of dentition rarely occurs. There is generally a condition of nervous excitation attendant upon the teething period, which in many cases is so slight as to express itself only as a somewhat increased nervous irritability in the child, productive of wakefulness, etc. Or the nervous irritation may be so increased in degree as to cause the most alarming and even fatal consequences. The period of teething is generally made manifest by this increased nervous irritability, and an increased flow of saliva from the mouth. The gums may be more or less congested over the presenting teeth, the positions of which are usually clearly discernable by reason of the gum being elevated and made tense by the erupting tooth crown beneath. The tumefaction or congestion of the overlying gum may be entirely absent, even in cases where the most profound nervous manifestations are present, due wholly to the peripheral nervous irritation caused by the advancing tooth. It is this class of cases where the local manifestations are but slight, and the general disturbance is profound, that has been the cause of the controversy which has been waged around the question of gum lancing. Those who hold to the belief of Forchheimer deny that any relief of general systemic disturbances can follow the operation in such cases. Where they have to deal with a case presenting a congested condition of the gum tissues, they admit that the operation may be useful by letting blood, but no further. The explanation of this belief is not far to seek—it is due to the fact that they understand the operation of gum lancing to mean a superficial scarification of the gum tissue, to empty the congested vessels of the parts and so reduce the local hyperæmia.

I have taken some pains to investigate the matter when opportunity has offered, and I have never found an operator who objected to gum lancing who did not have exactly this conception of the operation. This is gum scarification and not gum lancing. Gum lancing is a totally different procedure, undertaken not for the relief of congestion; of the gum, but for the purpose of freeing the tooth from restraint by the unyielding gum which covers it, causing backward pressure of the undeveloped tooth root upon the formative dentinal papilla at its base; the irritation of this latter is the cause of the nervous disturbances which it is the purpose of the operation to relieve. The conditions which demand relief by gum lancing are so graphically told by the late Dr. J. W. White, in the *American System of Dentistry*, that I cannot do better than quote from him:

"The direct pressure of the advancing tooth upon the fibrous integuments is not the only nor the principal factor in disturbance of equilibrium in pathological dentition. The most serious complications are, it is reasonable to suppose, caused by the resistance of the gums, and consequent pressure upon the nervous and vascular supply of the pulp, giving rise to severe and unremitting pain—a true toothache, comparable only to that exquisite torture which is experienced in after life from an exposed and irritated pulp. The condition, when a tooth is thus situated, is not unlike that which is found in whitlow, vascular and sensitive tissues bound down by unyielding coverings. If such a perversion of this physiological process is possible, there can be no question as to the extent of the mischief which may result—an irritability of the general system which finds expression in loss of appetite, sleeplessness, nausea, thirst, fever, diarrhœa or constipation, convulsions, paralysis, and other serious lesions, many of which, as strabismus or epilepsy, remain throughout life."

If, then, morbid symptoms coincident with the teething period manifest themselves, and their history and character point to a dental origin, the operation of dividing the gum over the presenting tooth should be so performed that the crown shall be completly freed from its imprisonment by the overlying tissues. It is frequently necessary to include in the operation not only the teeth immediately presenting, but those next in order of eruption in each jaw. If the operation has been properly done, it should be followed almost immediately by marked improvement in the general condition of the infant, and instant relief to the nervous distress.

The technique of the operation is quite simple. The child should be placed on a pillow lengthwise, supported on the lap of the nurse or assistant, seated on a chair facing the operator, and with the back toward the source of light, which should come preferably from a north window. The operator seats himself in front of the nurse, with the end of the pillow supporting the child's head in his lap. He then has command of the territory of operation, and can, by holding the child's head, guard against any sudden movement. The hands and body of the child are to be firmly held by the assistant. The best form of lancet for the operation is a small curved bistury, such as is sold at the depots for the purpose, but with the needle-like point ground off to a small but keen, rounded edge. The lancet is to be passed through the overlying tissue until it is felt to come into contact with the enamel surface, and the tissue divided a sufficient distance to allow the tooth to erupt without resistance.

For the incisors a single linear cut along the incisive edge is sufficient; for the cuspids and molars, a crucial incision is required. The operation is not excessively painful, and the pain is reduced to a minimum when a properly sharpened knife is used dexterously. Little hemorrhage follows, but if persistent, some slight styptic, such as powdered alum or phenol sodique may be used.

Nearly all medical writers agree that the teething period is one fraught with danger. Statistics show that the percentage of infant mortality is markedly higher during teething. A long series of infantile disorders occur most frequently during that period, and while recognizing this coincidence we may find many otherwise intelligent practitioners ignoring the possibility of a casual relationship between these diseases of infancy and the teething process, and, consequently, condemning the operation of gum lancing, not only as useless, but dangerous.

A recent published work on "Diseases and Injuries of the Teeth," by Messrs. Morton Smale aud J. E. Coyler, of London, contains the following suggestive statement: "Many healthy children pass through this period without any untoward symptoms, but many succumb, as may be gathered from the tables of mortality, teething being the cause of over 4.8 per cent. of deaths in children under 12 months, and 7.8 per cent. between the ages of 1 and 3 years." These same authors, however, notwithstanding this statement, are inclined to regard gum lancing as not useful save as a blood-letting measure. I have been unable so far to find any reported case of fatal result from gum lancing, nor have I knowledge of any untoward result occasioned by it when correctly performed. It is quite true that no precise scientific demonstration by the microscope or by post-mortem examination has been made to settle the question of the exact rationale of the procedure pro or con. The conditions are such that it perhaps never can be made, but this same objection might be as potently urged against many other well-established therapeutic measures in constant legitimate use.

The value of gum lancing in difficult dentition is established almost solely on clinical evidence, though it is difficult to understand why the perfectly plausible hypothesis of the rationale of its action should be rejected by its opponents as imperfect, when that set up by them in rebuttal is so manifestly illogical and inconsistent. Clinical evidence depends for its value upon the character and relationships of the phenomena observed, and the frequency with which certain related phenomena repeat themselves under similar conditions. If, for instance, infantile convulsions occurring coincidently with difficult or delayed eruption of the teeth are found to be relieved by a judicious use of the gum lancet, and a favorable result is obtained invariably in a number of cases so treated, we should be justified in assuming the casual relation between difficult dentition and infantile convulsions within certain limits, and be justified in the use of the lancet as a therapeutic measure for their relief. This relationship has been repeatedly observed. I have in several instances seen teething children, where convulsive seizures had supervened until the child was almost comatose, relieved at once and veritably snatched from the jaws of death by freely dividing the gum over the retarded teeth.

But convulsive seizures are not the only pathological result of delayed dentition. The irritation caused by the advancing tooth is but slight at first, and extends over a considerable period of time. The impress upon the nervous system of the child may be comparatively slight, so that its expression may not be manifested in the explosive outbursts of the nervous system which we know as convulsions. The nervous stress is more commonly manifested in loss of appetite, impairment of the digestive function, and nausea. Impairment of the digestive function, due to interference with the innervation of the stomach, whereby he food ingested becomes itself a source of irritation through the establishment of fermentative process throughout its mass, leads to and is accountable for the train of intestinal disorders, infantile diarrhœas, intestinal catarrhs, etc., which so oftrn accompany the teething process, and constitute the *bete noir* of mothers in rearing their children through the much dreaded second summer. Where the digestive sequelæ of pathological dentition have established themselves, the lancet cannot be expected to effect a cure unaided. Its use should be followed by appropriate constitutional treatment.

The close relationship of difficult dentition and capilarly bronchitis in infants has frequently been noted by medical writers and practitioners, but the idea of pathological dentition is as predisposing, not to say exciting, cause seems to have been overlooked until recently, notwithstanding the fact that in very many of the fatal cases of crupous pneumonia in many children there has been a definite history of difficult dentition immediately antecedent to the pulmonary attack. Recent observations lead me to suspect that in these cases the antecedent condition of difficult or pathological dentition has been the cause which induced the subsequent attack of capillary bronchitis.

In this connection it may be well to call to your attention the investigations recently published by Dr. Emil Schreier, of Vienna, with respect to the nature of the infecting organism in apical pericementitis. This observer found in all the apical inflammation about the roots of teeth which he examined, twenty or more in all, the diplococcus pneumoniæ, invariably present as the exciter of the inflammatory process. This is in line with Miller's investigations, which showed that the diplococcus pneumoniæ was a constant inhabitant of the mouth. As a further link in this particular chain of evidence, Dr. C. N. Peirce recently recounted to me two cases of incipient croupous pneumonia which occurred at different times in a family who were patients of his. In each instance the child was suffering from difficult eruption of the teeth, and in each case croupous pneumonia was set up as a sequeal. In both cases Dr. Peirce performed gum lancing, and in both cases there was subsidence of all the distressing symptoms in a few hours, with rapid and complete recovery. To any one who has investigated this subject, especially from the clinical standpoint, there can be no doubt as to the great utility of the operation in relieving in many cases the most alarming symptoms. It is simply and easily performed, and there are no weighty objections which can be urged against it, so that it is a matter of continued wonder that there can be found in the ranks of medical practitioners those who still strongly oppose it.

It may be asked of what interest can the question be to the dentist, who is seldom, if ever, called upon to operate in these cases. To this I would answer, prepare yourselves by an intelligent understanding of the operation and its correct relationships, and you will be consulted with respect to these matters when it is known that you are competent to give judgment upon them. Or if the knowledge has no usefulness outside of your immediate family, it may still afford you the opportunity to save the life of some one dear to you, as I verily believe it has upon more than one occasion in my own family circle.—*Practitioner and Adveriser.*

